# Morphology and Properties of Mg_2_Si Phase Modified by Pb in As-Cast Mg-2.5Si-*x*Pb Alloys

**DOI:** 10.3390/ma17081811

**Published:** 2024-04-15

**Authors:** Liang Chen, Wenpeng Yang, Hongbao Cui, Ying Wang, Zhichao Xu

**Affiliations:** 1 School of Materials Science and Engineering, Henan Polytechnic University, Jiaozuo 454000, China; cl721@hpu.edu.cn (L.C.); cuihongbao@hpu.edu.cn (H.C.); lxcwy@hpu.edu.cn (Y.W.); xzc@hpu.edu.cn (Z.X.); 2 School of Physics and Electronic Information Engineering, Henan Polytechnic University, Jiaozuo 454000, China; 3 Henan International Joint Research Laboratory for High-Performance Light Metallic Materials and Numerical Simulations, Jiaozuo 454000, China

**Keywords:** Mg_2_Si, Mg_2_(Si*_x_*Pb_1−*x*_) phases, modification, morphology, first-principles calculation

## Abstract

Pb plays an important role in determining the morphologies and mechanical properties of the Mg_2_Si phase in Mg-2.5Si-*x*Pb alloys. As the amount of Pb increases from 0.4 wt.% to 1 wt.%, the primary Mg_2_Si phase is refined during solidification. Its morphologies transform from equiaxed-dendrite to polygonal and finally to roughly circular. The key reason for morphology evolution is the preferential adsorption of Pb atoms on Mg_2_Si {100} surfaces to suppress the growth rate along the ⟨100⟩ directions, which is demonstrated by the adsorption model based on first principles. In addition, the hardness of the Mg_2_Si phase decreases with the increasing solution content of Pb according to the results of the nanoindentation. With the addition of Pb at 1 wt.%, Pb content in the primary Mg_2_Si phase reaches a maximum of 0.4 wt.%, and the hardness of the primary Mg_2_Si phase reaches a minimum of 3.64 GPa. This reduction in hardness is attributed to the augmented ionic bond ratio resulting from the solution of Pb, which concurrently enhances the toughness of the Mg_2_Si phase.

## 1. Introduction

Magnesium alloys are commonly utilized as cast components in aerospace and automotive applications [1,2] owing to their advantageous properties, including lightweight, high stiffness, specific strength, and exceptional damping capacity [3,4]. However, a significant limitation of traditional magnesium alloys is their reduced strength at temperatures exceeding 400 K, which restricts their broader applicability [5]. Consequently, there exists an urgent need to develop cost-effective high-temperature magnesium alloys suitable for use in structural components [6].

To enhance the mechanical properties of magnesium alloys at elevated temperatures, several strategies can be employed. These include incorporating strengthening particles with high thermal stability, reducing the element diffusion rate within the magnesium matrix, and optimizing both the grain-boundary structure and the overall microstructure [7]. Mg-Si alloys stand out as an ideal choice for large-scale commercial heat-resistant applications due to their simple production process and low cost. The core heat-resistant strengthening phase of Mg-Si alloys is Mg_2_Si, which exhibits lots of exceptional mechanical properties and thermal stability [8]. It can improve mechanical properties and reduce the creep rate of Mg alloy at high temperatures by inhibiting grain-boundary sliding. However, the Mg_2_Si phase in Mg-Si alloys tends to form coarse dendrites in the traditional metallurgical process [9]. The presence of coarse and brittle Mg_2_Si particles affects the mechanical properties of Mg-Si alloys, specifically the strength and ductility [10,11]. Recently, extensive research has been conducted to control the morphologies and size of the Mg_2_Si phase by adding elements [12,13,14,15] and compounds [16] or employing external high-intensity physical energy fields [17]. While these approaches have led to improvements in mechanical properties, there remains a significant gap in research aimed at enhancing the brittleness of Mg_2_Si while maintaining its inherent thermal stability. As a result, cracks often initiate within primary Mg_2_Si particles, compromising the structure of Mg alloys [18]. Consequently, it is crucial to tackle this issue by enhancing the toughness while controlling the morphology and size of the Mg_2_Si phase in order to fully realize the potential benefits of Mg_2_Si reinforcement.

One promising approach is through the substitution solid solution. For instance, Wang et al. [19] found that the plasticity of Sn-doped Mg_2_Si is superior to that of a pure Mg_2_Si phase. Pb, as a member of the IVA family in the periodic table alongside Si, exhibits the lowest electronegativity among elements within this group [20]. The increase in the electronegativity difference between bonded elements enhances the ionic nature of the bond, leading to improved toughness and plasticity of the material [21]. Additionally, the intermetallic compound Mg_2_Pb has a lattice structure similar to that of Mg_2_Si, facilitating the good solubility of Pb/Si in Mg_2_Si/Mg_2_Pb [22]. The solid solution of Pb atoms could change the bonding characteristics of the Mg_2_Si crystal. The change in the binding properties of these crystals could significantly affect the morphology and mechanical properties of the crystal [23]. Consequently, Pb is a potential ideal additive; it is anticipated to enhance the toughness of the Mg_2_Si phase while refining the morphology of Mg_2_Si particles. Furthermore, compared to rare earth elements, Pb is a more economical material. Although Pb vapor has health risks, the addition of a small amount with proper care will not be serious one [24]. In order to reveal the effects of Pb on the Mg_2_Si phase and the strengthening potentials of the Mg_2_(Si*_x_*Pb_1−*x*_) phase, first-principles calculations were employed to analyze the modification mechanism of the Mg_2_Si phase. Based on these calculations, further investigations were conducted on the morphologies and mechanical properties of the Mg_2_Si phase.

## 2. Theoretical Model, Calculation Method, and Experimental Procedures

### 2.1. Theoretical Model and Calculation Method

**Figure 1:**
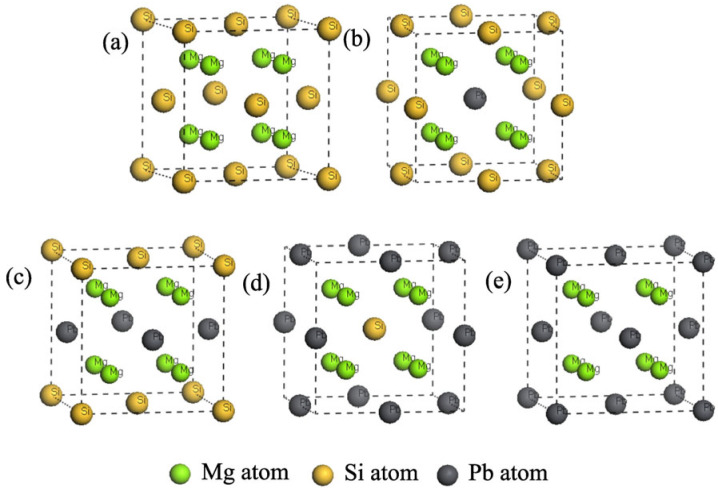
Crystal structures of Mg_2_(Si*_x_*Pb_1−*x*_), *x* = (**a**) 1.00, (**b**) 0.75, (**c**) 0.5, (**d**) 0.25, (**e**) 0.

Mg_2_Si and Mg_2_Pb have an anti-fluorite crystal structure, characterized by a space group of Fm$\bar{3}$m and a space number of 255. The lattice constants for Mg_2_Si and Mg_2_Pb are 0.6338 nm and 0.6933 nm [25], respectively. The unit cell atom coordinates are precisely as follows: the Mg (8c) position is at (1/4, 1/4, 1/4), while Si/Pb (4a) occupies (0, 0, 0). Based on previous studies [22], the doping of Pb atoms in the Mg_2_Si phase is performed by substituting Si atoms with Pb atoms to form a substantial solid solution Mg_2_(Si*_x_*Pb_1−*x*_). The Mg_2_(Si*_x_*Pb_1−*x*_) phase retains the anti-fluorite crystal structure across varying *x* values, as shown in Figure 1.

Computational analyses were conducted using the plane-wave pseudopotential density functional theory (DFT) method embedded in the CASTEP package (Materials Studio 7.0). The calculations employed an ultra-soft pseudopotential alongside the generalized gradient approximation PBE scheme. The plane-wave cutoff energy was fixed at 400 eV, and the *k*-point meshes were set to 11 × 11 × 11. First, the cell model of Mg_2_(Si*_x_*Pb_1−*x*_) was geometrically optimized using the BFGS algorithm. Subsequently, self-consistent iterative SCF calculations were performed based on the most stable Mg_2_(Si*_x_*Pb_1−*x*_) crystal structure, with electron relaxation handled via the Pulay density mixing method. Convergence criteria included a self-consistent field tolerance of 1 × 10^−6^ eV/atom and a maximum stress limit of 0.05 GPa. The total energy finally converged to less than 5 × 10^−6^ eV/atom. The valence states involved comprised Mg 3*s*^2^2*p*^6^, Si 3*s*^2^3*p*^2^, and Pb 5*d*^10^6s^2^6*p*^2^.

### 2.2. Experimental Method

To prepare Mg-2.5%Si-*x*%Pb (*x* = 0, 0.4, 0.8, 1; wt.%) alloys, commercial pure Mg (99.7 wt.%) and Mg-10 wt.%Si master alloys were melted at 780 °C in an electric-resistance furnace. A Pb piece was added, with different amounts to the melt. After stirring 3 times with a mean time interval of 15 min, the melt was held for 30 min at 780 °C and then cast into a graphite mold, which was preheated to 400 °C.

For metallographic analysis, specimens underwent mechanical grinding and polishing, followed by a 3 s etch in a Nital solution (4 vol.% HNO_3_, 96 vol.% C_2_H_5_OH). An OLYMPUS optical microscope (OM) (Olympus Corporation, Tokyo, Japan) and a Merlin Compact filed emission scanning electron microscope (SEM) (Carl Zeiss, Oberkochen, Germany) equipped with an Oxford Instruments (Abingdon, UK) energy-dispersive X-ray spectrometer (EDS) were used to examine the microstructures. Transmission electron microscope (TEM) specimens, 3 mm in diameter, were ground to about 60 µm and then, twin-jet electro-polished in a −30 °C solution of 15% nitric acid and 85% methanol. Before TEM observation, the surface oxide film was removed by ion-milling with a Fischoine model 1010 (Fischione Instruments, Cleveland, OH, USA) at 5 kV. TEM and HRTEM observations were conducted on a JEM-2100 microscope (JEOL Ltd., Tokyo, Japan) at 200 kV. TEM and HRTEM observations were carried out in a JEM-2100 microscope operating at 200 kV. Nanomechanical testing was performed on a Hysitron TI-Premier nanoindenter (Bruker, Billerica, MA, USA) equipped with a Berkovich diamond tip of a three-sided pyramid Berkovich probe (Bruker in USA). A trapezoidal loading profile was used to determine the nanomechanical properties of the Mg_2_(Si*_x_*Pb_1−*x*_) phase, which consisted of 5 s of loading, 2 s hold at 1000 μN, and 5 s of unloading.

## 3. Results

### 3.1. Structural Stability of the Mg_2_(Si_x_Pb_1−x_) Phase

The optimized lattice parameters of the Mg_2_(Si*_x_*Pb_1−*x*_) phase are listed in Table 1. The calculated lattice parameters of the optimized Mg_2_Si and Mg_2_Pb are consistent with the experimental data, with an error of about 0.43% and 0.07%, respectively. The lattice constant *a* of Mg_2_(Si*_x_*Pb_1−*x*_) (*x* ≠ 0, 1) falls within the range of the constants of the Mg_2_Si and Mg_2_Pb phases.

**Figure 2:**
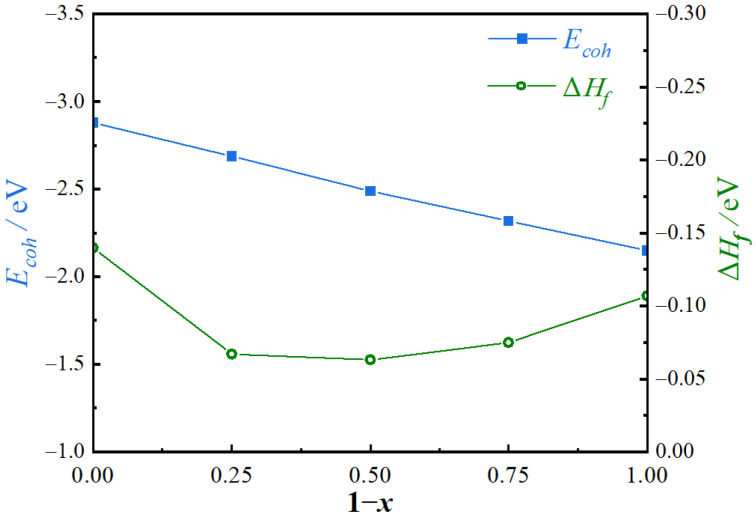
Formation enthalpies and cohesive of Mg_2_(Si*_x_*Pb_1−*x*_) phases.

The structural stability of a crystal is associated with its formation enthalpy per atom (Δ*H*_f_) of per atom and cohesive energy (*E*_coh_). The formation enthalpy quantifies the level of difficulty in forming an intermetallic compound by measuring the amount of energy released or absorbed during substance reactions [28]. On the other hand, cohesive energy measures the work performed by external forces when a crystal disintegrates into individual atoms [29]. The formation enthalpies of per atom (Δ*H*_f_) and cohesive (*E*_coh_) were calculated by the following formulae.

(1) $$\Delta H_{f}=\frac{1}{n+m+k}(E_{\mathrm{tot}}-nE_{\mathrm{solid}}^{\mathrm{Mg}}-mE_{\mathrm{solid}}^{\mathrm{Si}}-kE_{\mathrm{solid}}^{\mathrm{Pb}}),$$

(2) $$E_{\mathrm{coh}}=\frac{1}{n+m+k}(E_{\mathrm{tot}}-nE_{\mathrm{atom}}^{\mathrm{Mg}}-mE_{\mathrm{atom}}^{\mathrm{Si}}-kE_{\mathrm{atom}}^{\mathrm{Pb}}),$$

where *E*_tot_ is the total energy of Mg_2_(Si*_x_*Pb_1−*x*_), $E_{\mathrm{solid}}^{\mathrm{Mg}}$, $E_{\mathrm{solid}}^{\mathrm{Si}}$, and $E_{\mathrm{solid}}^{\mathrm{Pb}}$ are the energies per atom of Mg, Si, and Pb, respectively. $E_{\mathrm{atom}}^{\mathrm{Mg}}$, $E_{\mathrm{atom}}^{\mathrm{Si}}$ and $E_{\mathrm{atom}}^{\mathrm{Pb}}$ are the energies of isolated atoms for each element. *n*, *m,* and *k* are the numbers of Mg, Si, and Pb atoms in a unit cell, respectively. The formation enthalpies (Δ*H*_f_) and cohesive (*E*_coh_) of the predicted phase for different compositions (1 − *x*) are shown in Figure 2.

A negative formation enthalpy indicates an exothermic reaction, while a low formation enthalpy signifies robust structural stability [25]. The negative formation enthalpy of each Mg_2_(Si*_x_*Pb_1−*x*_) phase suggests that the stability of these phases remains before and after solid solution in the system, thereby indicating their potential for stable formation. Mg_2_Si exhibits the lowest formation enthalpy, while Mg_2_Pb possesses a slightly higher formation enthalpy compared to Mg_2_Si but lower than other Mg_2_(Si*_x_*Pb_1−*x*_) phases (*x* ≠ 0, 1). It can be inferred that, compared to Mg_2_Si and Mg_2_Pb, the formation ability of the Mg_2_ (Si*_x_*Pb_1−*x*_) phase is relatively limited. Furthermore, a low cohesive energy is indicative of elevated structural stability [20]. As can be seen in Figure 2, the negative value of the cohesive energy decreases with the increasing Pb content. It indicates that the most stable composition is the Mg_2_Si phase, and the least is the Mg_2_Pb phase, in Mg_2_(Si*_x_*Pb_1−*x*_) phases with different compositions.

### 3.2. Elastic Constants and Mechanical Properties of the Mg_2_(Si_x_Pb_1−x_) Phase

The elastic constant *C_ij_* serves as an indicator of a material’s resistance to external forces and deformations. It is determined by analyzing the relationship between stress and strain [20]. For its cubic symmetry, Mg_2_(Si*_x_*Pb_1−*x*_) have three independent elastic constants: *C*_11_, *C*_12,_ and *C*_44_. Combined with the Voigt–Reuss–Hill approximation, the bulk moduli *B*, shear moduli *G*, Young’s moduli *E*, and Poisson ratio *ν* can be deduced by the following formulae, respectively [30]:

(3) $$B=\frac{C_{11}+2C_{12}}{3},$$

(4) $$G=\frac{C_{11}-C_{12}+3C_{44}}{5},$$

(5) $$E=\frac{\left(C_{11}-C_{12}+3C_{44})(C_{11}+2C_{12}\right)}{3C_{11}+2C_{12}},$$

(6) $$\nu =\frac{3B-E}{6B},$$

The elastic constants computed for the Mg_2_(Si*_x_*Pb_1−*x*_) phases are presented in Table 2. It was evident that Mg_2_(Si*_x_*Pb_1−*x*_) phases satisfied the mechanical stability requirements specific to cubic crystal systems, including *C*_44_ > 0, *C*_11_ + 2*C*_12_ > 0, and *C*_11_ − *C*_12_ > 0 [31]. Among Mg_2_(Si*_x_*Pb_1−*x*_) phases with different Pb contents, the Mg_2_Si phase exhibits the highest Young’s modulus of 102.00 GPa, the highest shear modulus of 43.35 GPa, and the lowest Poisson’s ratio of 0.1763. As the solid solubility of Pb increases, the Young’s and shear moduli for Mg_2_(Si*_x_*Pb_1−*x*_) phases decrease significantly, while the Poisson’s ratio increases.

Furthermore, the hardness can be predicted through the first-principles calculations utilizing the following formulae [32]:

(7) $$H_{V}={\left[\prod^{x-y}(H_{V}^{x-y})n^{\left(x-y\right)}\right]}^{1/\sum n^{x-y}},$$

(8) $$H_{v}^{x-y}=\frac{350{\left(N_{e}^{x-y}\right)}^{\frac{2}{3}}e^{-1.191f_{i}^{x-y}}}{{\left(d^{x-y}\right)}^{2.5}},$$

(9) $$f_{i}^{x-y}=1-e^{-\frac{\left|p_{c}-p\right|}{p}},$$

(10) $$N_{e}^{x-y}=\frac{\left(\frac{z_{x}}{N_{x}}+\frac{z_{y}}{N_{y}}\right)\left[\sum n^{x-y}{\left(d^{x-y}\right)}^{3}\right]}{{\left(d^{x-y}\right)}^{3}V},$$

where $H_{V}^{x-y}$, $n^{x-y}$, $d^{x-y}$, $f_{i}^{x-y}$ are the hardness, bond number, bond length, and Phillips ionicity of the *x-y* bonds, respectively. *P* is the overlap population of a bond, and *Pc* is the overlap population of a bond in a hypothetical pure covalent crystal with the same special structure (*P_c_* = 0.75). *Z_x_* or *Z_y_* is the valence electron number. *N_x_* or *N_y_* is the coordination number of the *x* or *y* atom constructing the *x-y* bond. *V* is the volume of the calculating unit cell. The calculated bond parameters and Vickers hardness values are shown in Table 3.

Generally, covalent bonds contribute to high strength and hardness, while ionic bonds are associated with high toughness. The ratio of covalent and ionic bonds in the Mg_2_(Si*_x_*Pb_1−*x*_) phase can be determined by Muliken’s overlap population *P*. A value of *P* = 0 indicates purely ionic bonding, whereas *P* > 0 suggests an increase in covalent action [33]. According to the calculation results, the binding between atoms in the Mg_2_(Si*_x_*Pb_1−*x*_) phase occurred mainly through covalent bonds with a minor presence of ionic bonds. With increasing Pb content, the proportion of covalent bonds decreased while the percentage of ionic bonds increased. Consequently, the trend of hardness aligns with that of the Young’s and shear moduli (Table 2), whereby an increase in the solid solubility of Pb in the Mg_2_Si phase led to a decrease in the hardness of the Mg_2_(Si*_x_*Pb_1−*x*_) phase. This change enhances the ductility and toughness and reduces the brittleness of the Mg_2_Si phase.

### 3.3. Growth Morphology of Mg_2_(Si_x_Pb_1−x_) Crystal

**Figure 3:**
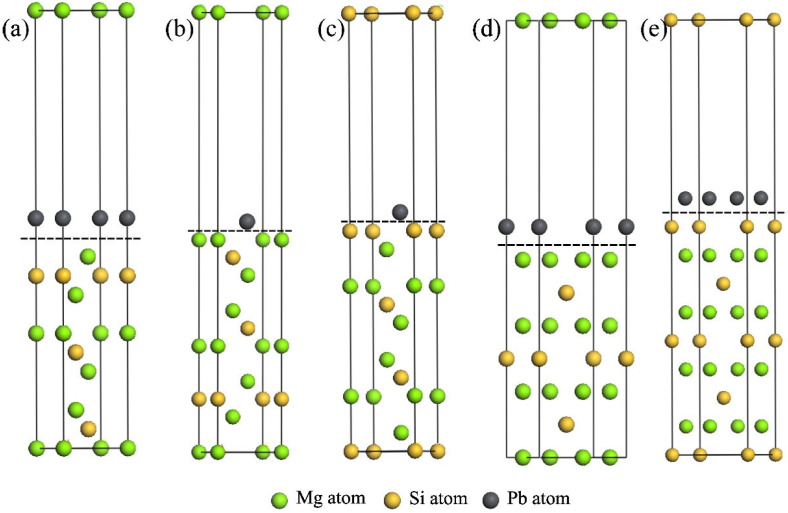
The surface-slab models for {111} and {100} faces of Mg_2_Si crystal. (**a**) Mg_2_Si {111}Mg-I termination, (**b**) Mg_2_Si {111}Mg-II termination, (**c**) Mg_2_Si {111}Si termination, (**d**) Mg_2_Si {100}Mg termination, (**e**) Mg_2_Si {100}Si termination. The dashed line in the figure represents the interface.

The formation process and final morphology of the primary Mg_2_Si phase are influenced by its inherent crystalline structure as well as external factors, including temperature, pressure, and solvent concentration [34]. From a crystallographic perspective, the {111} facets of Mg_2_Si, which exhibit the highest degree of surface packing, manifest the minimum surface energy, while the {100} facets have the lowest degree of packing and the highest surface energy. During crystal growth, the {111} faces expose, and the {100} faces undergo shrinkage [35]. The substitution of Si with Pb in Mg_2_Si crystal resulted in a modification of the surface energy, ultimately leading to a morphological transformation. The surface-slab models for the {111} and {100} faces were constructed to study the surface energy and adsorption capacity, respectively, as shown in Figure 3. The surface energy (*E*_surf_) of {111} and {100} faces, as well as the effect of substituting Si atoms with Pb atoms in all slab models, were determined. The corresponding calculation results are shown in Table 4. In pure Mg_2_Si, the surface energy of {111}Mg-I termination was the lowest, with a value of 0.48 eV. Upon substitution of Pb for Si, the surface energy of each termination increased. The {111}Mg-I and {111}Mg-II terminations exhibited a significant rise in surface energy, by 1.27 times and 1.21 times, respectively. The surface energy of {111}Mg-II termination became the highest of all terminations.

**Figure 4:**
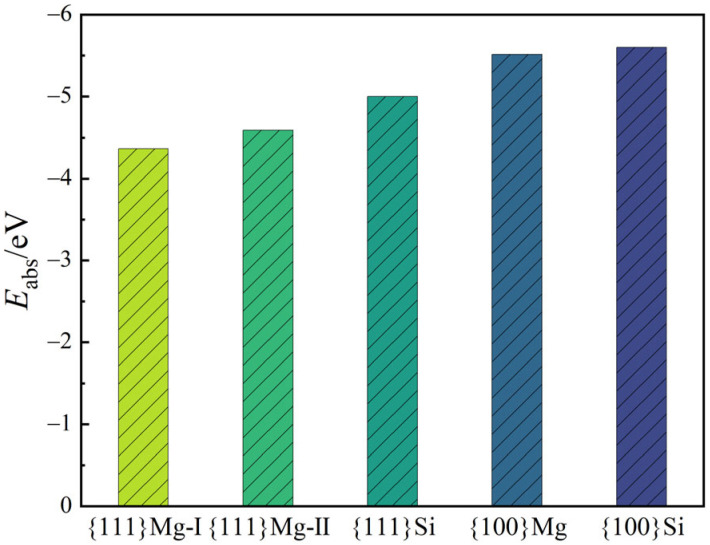
The absorption energies of Mg_2_Si {111} and {100} planes.

From the perspective of external growth conditions, the adsorption or bonding of Pb atoms in the liquid phase also exerts an influence on the growth rate of Mg_2_Si crystal faces. To illustrate this phenomenon, an adsorption model was established based on the replacement of Si sites by Pb atoms (as depicted in Figure 3). The adsorption energy *E*_ads_ of {111} and {100} faces for Pb atoms of all terminations are shown in Figure 4. Generally, a more negative adsorption energy indicates a stronger adsorption [36]. Consequently, Pb atoms exhibit a preference for adsorbing onto {100} faces.

### 3.4. Microstructure of Mg_2_(Si_x_Pb_1−x_) in Mg-Si-Pb Alloy

**Figure 5:**
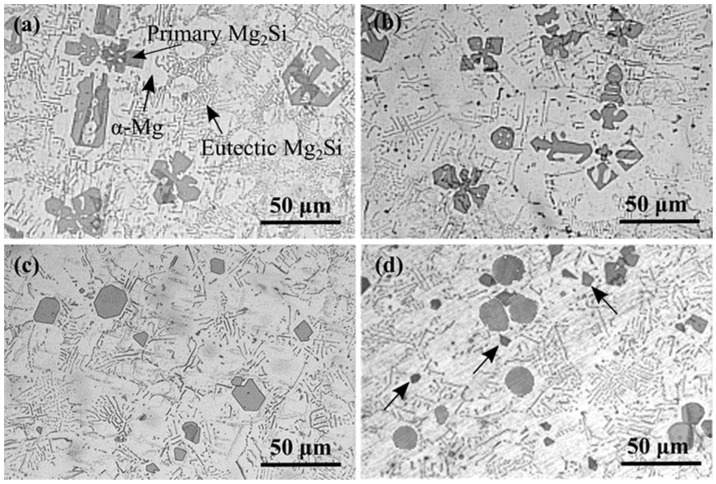
Optical microstructures of as-cast Mg-2.5Si alloys with different contents of (**a**) 0, (**b**) 0.4, (**c**) 0.8, (**d**) 1.0 wt.% Pb, respectively.

The microstructures of as-cast Mg-2.5Si-*x*Pb alloys are shown in Figure 5. Mg-2.5Si-*x*Pb alloys consisted of the Mg_2_Si phase and α-Mg matrix. In Mg-2.5Si-0.4Pb alloy (Figure 5b), the primary Mg_2_Si phase had a dendritic morphology, similar to that of Mg-2.5Si alloy (Figure 5a). However, there was an increase in the number of primary Mg_2_Si particles and a decrease in their size. The average particle size decreased from 50.1 μm in Mg-2.5Si alloy to 34.8 μm in Mg-2.5Si-0.4Pb alloy. With the increase of Pb content in the alloy, the morphology and size of the primary Mg_2_Si phase in the Mg-2.5Si-0.8Pb alloy changed significantly. As observed in Figure 5c, the morphology of the primary Mg_2_Si phase changed from equiaxed-dendrite to polygonal outlines, and the average particle size further decreased to 18.7 μm.

In Mg-2.5Si-1Pb alloy, the morphology of the primary Mg_2_Si phase exhibited a very special change. There was a significant difference in the size of primary Mg_2_Si particles (Figure 5d). Apart from particles with an average size of 16.9 μm, numerous finer particles with a size ranging from 3 to 6 μm were also present. The distinct characteristic was that while the larger Mg_2_Si particles had been spheroidized, the finer particles retained a polygonal shape. Nonetheless, a rounding effect was evident at the sharp corners of these fine particles, suggesting partial or incomplete spheroidization, as indicated by the arrows in Figure 5d.

Figure 5 shows high-magnification images of the microstructure. There was local energy fluctuation and compositional fluctuation during solidification, so the images seem to show an effect on eutectic Mg_2_Si and α-Mg. Multiple different areas of the alloys were measured to determine the proportion of each phase area, and the results are shown in Table 5. From the proportion of area in each alloy, it can be seen that the change of Pb content has little effect on the eutectic phase and α-Mg. This is due to the low Pb content in the alloy and the high solid solubility of Pb in Mg [22].

**Figure 6:**
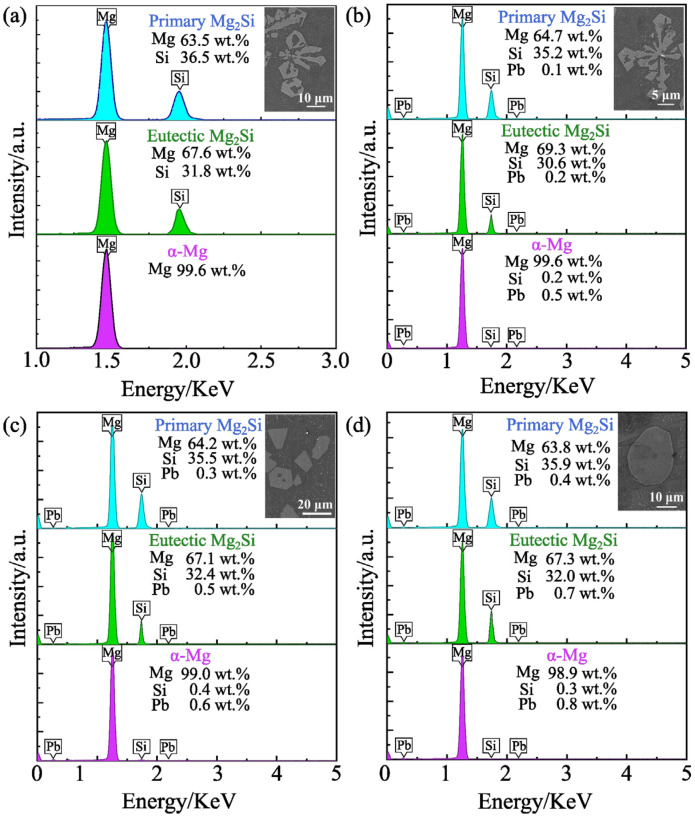
SEM micrograph and EDS results of Mg-2.5Si-*x*Pb alloy with different contents of (**a**) 0, (**b**) 0.4, (**c**) 0.8, (**d**) 1.0 wt.% Pb, respectively.

The distribution of alloying elements and the composition analysis of Mg-2.5Si-*x*Pb alloy were further examined using EDS, as shown in Figure 6. In Mg-2.5Si alloy (Figure 6a), the Si element was mainly distributed within the Mg_2_Si phase, and the Si content in both primary and eutectic Mg_2_Si phases was similar, with values of 36.5 wt.% and 31.8 wt.%, respectively. In Mg-2.5Si-0.4Pb alloy (Figure 6b), Mg_2_Si particles show a prominent Mg K_α1,2_ peak, a strong Si K_α1,2_, and a weak Pb L_α1,2_ peak. It is evident that the Si element is concentrated in the Mg_2_Si phase. The composition of the primary and eutectic Mg_2_Si phase was about Mg-35.2% Si-0.1% Pb and Mg-30.6% Si-0.2% Pb (wt.%), respectively. The Pb content within the Mg matrix was found to be 0.5 wt.%, which exhibited a significant increase compared to that present in the Mg_2_Si phase. This observation can be attributed to the high solid solubility of Pb in the Mg matrix.

**Figure 7:**
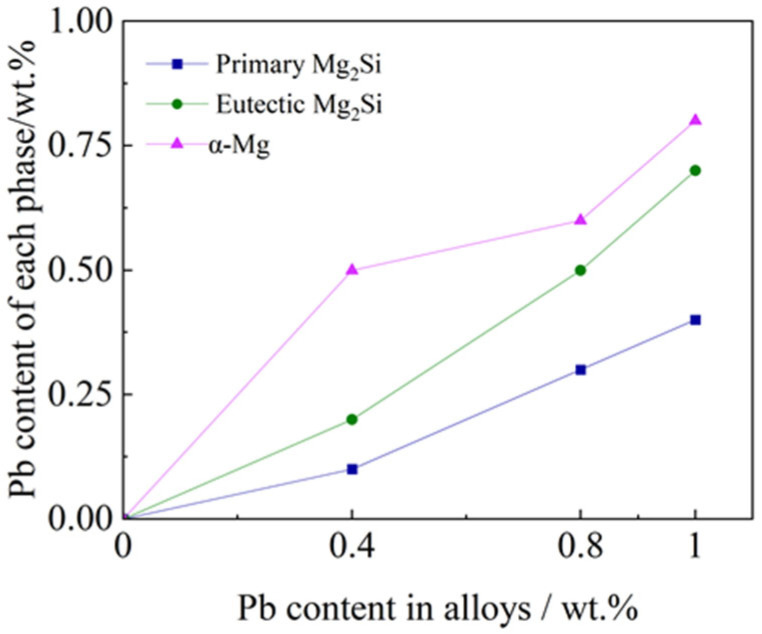
Pb content in each phase of as-cast Mg-2.5Si-*x*Pb.

The same analysis was performed on Mg-2.5Si-0.8Pb alloy (Figure 6c) and Mg-2.5Si-1Pb alloy (Figure 6d). In the EDS of primary and eutectic Mg_2_Si particles, a minor increase in peak intensity was noted for the Si and Pb elements. In Mg-2.5Si-0.8Pb alloy, the Pb content of primary and eutectic Mg_2_Si particles increased to 0.3 wt.%, and 0.5 wt.%, respectively. In Mg-2.5Si-1Pb alloy, the compositions of primary and eutectic Mg_2_Si were about Mg-35.9%Si-0.4%Pb and Mg-32.0%Si-0.7%Pb (wt.%), respectively. The variation in Pb content within the primary Mg_2_Si phase, eutectic Mg_2_Si phase, and α-Mg matrix for Mg-2.5Si-*x*Pb is shown in Figure 7. There was a consistent increase in the proportion of Pb mass within each phase with the increase of Pb content in the alloy. It is noteworthy that the Pb content detected in all phases was slightly smaller than that of the composition of the Mg-2.5Si-*x*Pb alloys. The possible reason was that the high density and atomic number of the Pb element make its absorption efficiency of X-ray and gamma-ray higher [37], resulting in a slight decrease in the measured Pb content. In addition, among all components of the alloy, the primary Mg_2_Si phase exhibited the lowest Pb content, and the Pb content in the Mg matrix was close to that in the nominal composition of the alloys. It is reasonable to conclude that the Pb content in the liquid phase had an important effect on the morphology of the primary Mg_2_Si phase. During the analysis of EDS, the influence range of the focused electron beam on the specimen was about 3 μm at 15 kV. Therefore, the content of Pb of the eutectic Mg_2_Si phase is between that in the primary phase and the Mg matrix. 

**Figure 8:**
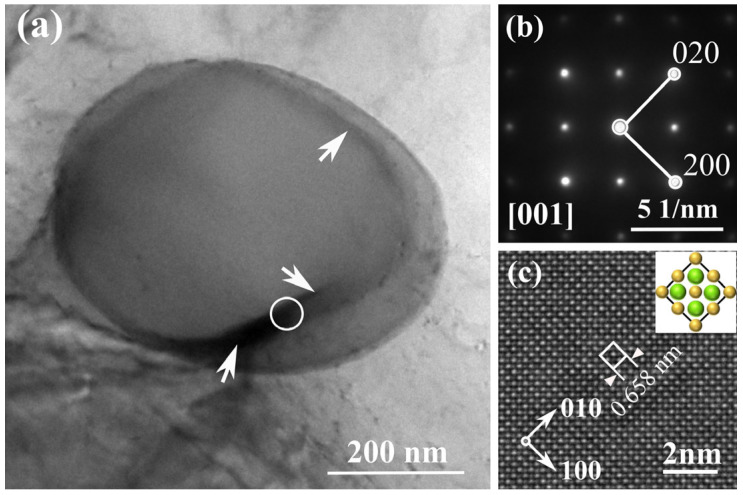
TEM micrographs of Mg-2.5Si-1Pb alloys. (**a**) TEM micrograph showing a primary Mg_2_(Si*_x_*Pb_1−*x*_) particle in Mg-2.5Si-1Pb alloy; (**b**) DP recorded from primary Mg_2_Si in (**a**); (**c**) HRTEM image showing the two-dimensional lattice structure of central region of the Mg_2_(Si*_x_*Pb_1−*x*_) phase in Mg-2.5Si-1Pb alloy and the inset showing the crystal structure of Mg_2_(Si*_x_*Pb_1−*x*_). The circle in panel (**a**) is the acquisition area of electron diffraction. Green balls represent Mg atoms, while yellow balls represent Si atoms in panel (**c**).

Mg-2.5Si-1Pb alloy, which has the highest Pb content, was selected for detailed investigation using TEM and HRTEM. Figure 8 shows TEM micrographs of a primary Mg_2_Si particle in Mg-2.5Si-1Pb alloy. The interior of the Mg_2_Si crystal was uniform. However, a distinct banded region with gray–black contrast was observed at the periphery of the Mg_2_Si particle, as indicated by white arrows. The electron diffraction pattern (DP) obtained from the [001]_Mg2Si_ zone axis of the circular region is shown in Figure 8b. HRTEM was further conducted on the edge region, as shown in Figure 8c. In this image, the gray and white columns correspond to the Si and Mg atomic columns, respectively. The inset highlighted the primary Mg_2_Si unit cell structure, which is outlined by squares. The measured value of crystal constant *a* was 0.658 nm and slightly larger than that of pure Mg_2_Si. The enlarged lattice constant can be ascribed to the substitution of Pb for Si. The edge region was thoroughly examined, and no atomic segregation was detected. Therefore, the presence of a gray–black banded area at the edge can be attributed to the thickness fringe due to the spherical morphology of this Mg_2_Si particle.

### 3.5. Nanomechanical Properties of the Mg_2_(Si_x_Pb_1−x_) Phase

**Figure 9:**
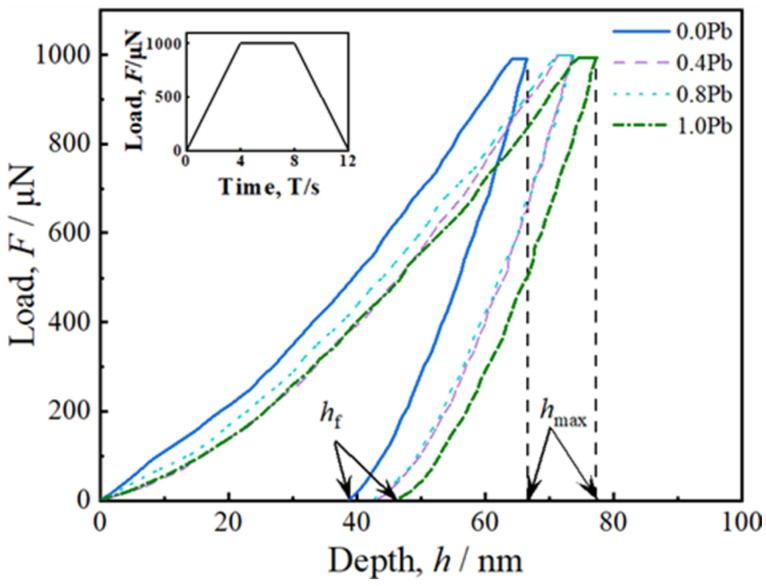
The load-displacement curves of primary Mg_2_(Si_x_Pb_1−x_) phase in Mg-2.5Si-*x*Pb alloys. *h*_max_ and *h*_f_ denote the maximum and residual indentation depth, respectively. An applied trapezoidal loading function is depicted in the inset.

Figure 9 presents the load-displacement curves obtained from in situ nanoindentation tests conducted on the primary Mg_2_Si phase in Mg-2.5Si-*x*Pb alloys. The maximum indentation depth *h*_max_ was 73.9 nm in the Mg-2.5Si-0.4Pb alloy and increased to 77.5 nm in Mg-2.5Si-1Pb. The value of plastic depth *h*_f_ increased from 42.9 nm to 45.8 nm.

The nanomechanical properties were calculated using the Oliver–Pharr method [38] based on the unloading segment data, as shown in Table 6. It is evident that the solid solution of Pb in the Mg_2_Si phase results in the decrease both of elastic modulus *E* and hardness *H*_v_. Among all compositions, the Mg_2_Si phase in Mg-2.5Si-1Pb alloy with the highest Pb content exhibits the lowest elastic modulus of 83.4 GPa and hardness of 3.6 GPa. Compared to the pure Mg_2_Si phase, there is an observed reduction of 11.8% in elastic modulus *E* and a decrease of 2.0% in hardness *H*_v_.

## 4. Discussion

### 4.1. Morphology

In FCC crystals, the preferential growth directions are ⟨100⟩, and {100} faces have the fastest growth rate [14]. Under ideal growth conditions, the {100} faces of Mg_2_Si crystal will gradually shrink during the growth process, ultimately degrading to corners and edges. This leaves the {111} facets exposed, resulting in an octahedral crystal shape [39]. The addition of Pb effectively modified the morphology of primary Mg_2_Si crystals in Mg-2.5Si-*x*Pb, primarily due to the changes in thermodynamics and kinetics conditions in front of the solid–liquid interface.

The incorporation of a few Pb atoms into primary Mg_2_Si crystals through substitution at the Si atoms resulted in a change of the surface energy of the {111} and {100} planes (Table 4). According to previous studies, the equilibrium form of an FCC crystal is determined by the proportional surface energies of the {100} and {111} planes [40]. With Si sites replaced by Pb atoms, the surface-energy ratio between the {100} Mg termination and {111} Mg-I termination changed from 2.23 to 1.21. Therefore, the morphology of primary Mg_2_Si crystals tended to be a truncated octahedron. Additionally, the calculation results of the adsorption energy of Pb atoms on the Mg_2_Si {111} and {100} planes (Figure 4) show the preferential adsorption of Pb atoms on {100} planes. The growth rates of ⟨100⟩ directions were inhibited, and the final morphology of the Mg_2_Si crystal was also affected.

Meanwhile, the growth rate was also apparently encouraged by large constitutional undercooling and supersaturation. Once the interface front of the primary Mg_2_Si was unstable, the main stem was formed along the preferential growth direction ⟨100⟩. Subsequently, secondary branches are aroused in directions perpendicular to the primary dendrite trunk. The rapid generation and growth of these secondary dendrites result in their interconnection or overlapping, as shown in Figure 5a. Due to the low Pb content in Mg-2.5Si-0.4Pb alloy, the primary Mg_2_Si phase also tends to form coarse dendrites, similar to those observed in Mg-2.5Si alloy. However, the addition of Pb induced a supercooling effect that significantly enhanced the nucleation capability of Mg_2_Si crystals, consequently leading to an augmentation in the quantity of primary Mg_2_Si particles and a reduction in size.

In Mg-2.5Si-0.8Pb alloy, the surface energy of Mg_2_Si crystal change caused by Pb substitution for Si, and the preferential adsorption of Pb atoms on the {100} plane became more obvious. The growth rates of ⟨111⟩ directions were hindered, resulting in the appearance of truncated octahedral morphologies. The reserved percentage of {100} facets was found to be correlated with the reduction in growth rates along the ⟨100⟩ directions [9].

**Figure 10:**
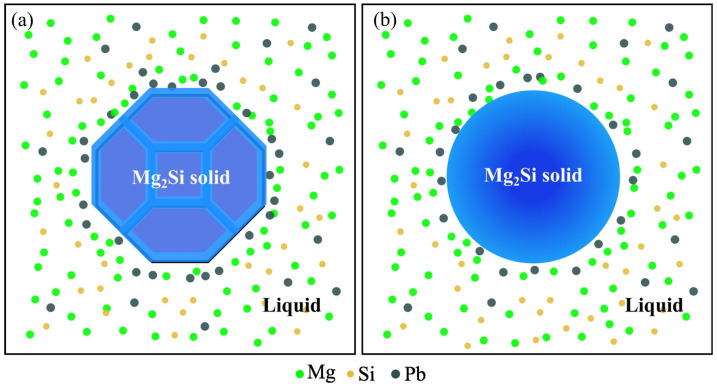
Schematic diagram of atomic distribution at the front edge of the solid-liquid interface of Mg_2_Si crystal (**a**) Mg-2.5Si-0.8Pb alloy; (**b**) Mg-2.5Si-1Pb alloy.

The formation of roughly spherical primary Mg_2_Si particles in the Mg-2.5Si-1Pb alloy (Figure 5d) was a complex process that requires further analysis. According to Mg-Si and Mg-Pb equilibrium phase diagrams [22], the primary Mg_2_Si crystals first nucleate and grow. However, due to solute trapping under non-equilibrium solidification, some Pb atoms were dissolved into Mg_2_Si, forming a substitution solid solution Mg_2_(Si*_x_*Pb_1−*x*_). According to TEM observations, Pb atoms only replaced Si atoms in Mg_2_Si crystal. In contrast, Sn not only has the capability to replace Si but also exhibits the potential for replacing Mg in Mg_2_Si crystal [41]. Therefore, the substitution solution probability of the Pb atom in the Mg_2_Si crystal is less than that of the Sn atom. Furthermore, the calculation results indicated that the Mg_2_Si structure exhibited the highest stability. Under ideal equilibrium conditions, the Mg_2_(Si*_x_*Pb_1−*x*_) phase had a greater tendency to decompose into Mg_2_Si and Pb in order to reduce the energy of the system. The above two reasons significantly restricted the solid solubility of Pb in the Mg_2_Si phase under conventional casting conditions. During the solidification process, the excess Pb atoms were expelled from the primary Mg_2_Si particle and gathered at the solid–liquid interface. At the sharp edge of two meeting growth planes, there was a notable increase in Pb concentration, as shown in Figure 10. Given that Pb has a higher atomic mass compared to Mg and Si [20], the diffusion rate of Pb atoms was considerably slower. The accumulation of Pb atoms at sharp corners hindered the transfer of atoms from the liquid to the solid phase, causing a decrease in growth rate at those locations. This promoted the morphological transformation of truncated octahedral Mg_2_Si particles into roughly spheroidal particles.

### 4.2. Mechanical Properties

According to the principles of general crystal-strengthening theoretical logic, the solubility of Pb in Mg_2_Si crystal is expected to result in the enhancement of solution strengthening, leading to an increase in the hardness of primary Mg_2_Si [42]. However, the nanomechanical properties data (Table 5) revealed contrary findings. The hardness of the Mg_2_(Si*_x_*Pb_1−*x*_) phase reduced with an increasing Pb solubility. The main reason was that the substitutional solid solution Mg_2_(Si*_x_*Pb_1−*x*_) belongs to a covalent compound. The hardness of the Mg_2_(Si*_x_*Pb_1−*x*_) phase is primarily determined by the energy of covalent bonds localized within electron spin pairs [43]. This factor remains unaffected by external influences such as impurities, precipitates, grain boundaries, and other related factors [44].

Examining the bond parameters listed in Table 3, it became evident that the covalent interaction of the Mg-Pb bonds (*P* = 0.24) was weaker than that of Mg-Si (*P* = 0.36) in the Mg_2_(Si_x_Pb_1−x_) crystal. Consequently, as the number of Pb atoms increased in the Mg_2_(Si_x_Pb_1−x_) crystal, the number of ionic bonds also increased. This led to a decrease in hardness and an increase in the toughness of the Mg_2_(Si*_x_*Pb_1−*x*_) phase. The mechanical properties of Mg_2_(Si*_x_*Pb_1−*x*_) predicted by the first-principles calculation (Table 2 and Table 3) aligned well with the experimental values. This agreement can be attributed to the fact that Pb atoms only replace Si atoms in Mg_2_Si (Figure 8). The formation of the substitution solid solution Mg_2_(Si*_x_*Pb_1−*x*_) was consistent with the crystal model established through first-principles calculations.

## 5. Conclusions

The morphology and mechanical properties of the Mg_2_(Si*_x_*Pb_1−*x*_) phase in the Mg-2.5Si-*x*Pb alloy were investigated through theoretical calculations and experimental analysis. The main conclusions are summarized as follows:

(i) Based on the first principles of structural prediction and electronic structure calculation, the Mg_2_(Si*_x_*Pb_1−*x*_) solid solution was structurally stable. The stability of Mg_2_(Si*_x_*Pb_1−*x*_) increased with the decrease in Pb content. Compared with the Mg_2_Si phase, Mg_2_(Si*_x_*Pb_1−*x*_) had sufficient hardness and inherent toughness as a reinforcement phase;

(ii) The preferential adsorption on {100} crystal planes of Pb atoms changes the growth rate along the 〈100〉 directions of the Mg_2_Si phase. As the Pb content in the Mg_2_(Si*_x_*Pb_1−*x*_) phase increased, the morphology of primary Mg_2_(Si*_x_*Pb_1−*x*_) transformed from equiaxed-dendrite to truncated octahedron, and then to, roughly, sphericity in the Mg-2.5Si-1Pb alloy;

(iii) As a substitutional solid solution, the Mg_2_(Si_x_Pb_1−x_) phase is formed due to the solute trapping during solidification. The stiffness and hardness of the Mg_2_(Si_x_Pb_1−x_) phase decreased with the increase of Pb content. The experimental findings regarding mechanical properties were found to be in accordance with the theoretical predictions obtained through first-principles calculations. Combined with the values of elastic modulus obtained from theoretical calculations, it can be seen that the solid solutions of Pb can reduce the brittleness and improve the toughness of the Mg_2_Si phase.

## Figures and Tables

**Table 1:** Lattice constants of Mg_2_(Si*_x_*Pb_1−*x*_).

*x*		1.00	0.75	0.50	0.25	0.00
*a* (nm)	Theoretical	0.6365	0.6623	0.6704	0.6775	0.6938
	Experimental	0.6338 [26]	-	-	-	0.6933 [27]

**Table 2:** Calculated elastic constants and moduli of the Mg_2_(Si_x_Pb_1−_) phase.

*x*	*C*_11_, GPa	*C*_44_, GPa	*C*_12_, GPa	*B*, GPa	*G*, GPa	*E*, GPa	ν	*G/B*
1	114.04	41.50	21.76	52.52	43.35	102.00	0.1763	0.83
0.75	92.29	35.21	19.32	43.64	35.72	84.19	0.1785	0.82
0.5	75.78	28.45	16.65	37.93	28.46	68.29	0.1999	0.75
0.25	70.93	26.99	23.09	39.04	25.76	63.35	0.2295	0.66
0	59.00	24.85	20.33	33.22	22.64	55.36	0.2223	0.68

**Table 3:** Calculated bond parameters and hardness of Mg_2_(Si*_x_*Pb_1−*x*_) phase.

Crystals	Bone Types	Number	*D* (Å)	*P*	*V* (Å^3^)	*H_v_* (GPa)
Mg_2_Si	Mg-Si	32	2.7562	0.36	257.8875	4.17
Mg_2_(Si_0.75_Pb_0.25_)	Mg-Si	24	2.8163	0.38	279.6076	3.03
	Mg-Pb	8	2.8780	0.03		
Mg_2_(Si_0.5_Pb _0.5_)	Mg-Si	16	2.8178	0.40	291.9998	2.84
	Mg-Pb	16	2.9284	0.10		
Mg_2_(Si_0.25_Pb_0.75_)	Mg-Si	8	2.8501	0.42	311.0440	2.69
	Mg-Pb	24	2.9629	0.18		
Mg_2_Pb	Mg-Pb	32	3.0022	0.24	333.2925	2.61

**Table 4:** The surface energies of different slab models of the Mg_2_Si phase.

Mg_2_Si	{111}Mg-I	{111}Mg-II	{111}Si	{100}Mg	{100}Si
*E*_surf_ (eV)	0.48	1.60	1.62	1.07	1.27
*E*_surf/Pb_ (eV)	1.09	3.53	1.65	1.32	2.79

**Table 5:** The area ratio of each phase of as-cast Mg-2.5Si-*x*Pb alloys.

Alloy	Primary Mg_2_Si (%)	Eutectic Mg_2_Si (%)	α-Mg (%)
Mg-2.5Si	17.5	3.5	78.2	
Mg-2.5Si-0.4Pb	17.6	3.8	77.7	
Mg-2.5Si-0.8Pb	18.6	4.8	76.3	
Mg-2.5Si-1.0Pb	19.9	4.6	75.1	

**Table 6:** The elastic modulus *E*, hardness *H*_v_, maximum indentation depth *h*_max_, plastic depth *h*_f_, and plastic deformation ratio *h*_f_/*h*_max_, of two different Mg_2_(Si*_x_*Pb_1−*x*_) phases.

Alloy	Elastic Modulus *E* (GPa)	Hardness *Hv* (Gpa)	*h*_max_ (nm)	*h*_f_ (nm)	*h*_f_/*h*_max_ (%)
Mg-2.5Si	94.6	4.6	66.8	38.7	57.9
Mg-2.5Si-0.4Pb	86.2	4.0	73.9	42.9	58.1
Mg-2.5Si-0.8Pb	83.7	3.9	73.7	41.8	56.7
Mg-2.5Si-1.0Pb	83.4	3.6	77.5	45.8	59.1

## Data Availability

Data are contained within the article.
